# Exploring Registered Psychiatric Nurses' Responses towards Service Users with a Diagnosis of Borderline Personality Disorder

**DOI:** 10.1155/2012/601918

**Published:** 2012-04-08

**Authors:** Bridget McGrath, Maura Dowling

**Affiliations:** ^1^HSE West, Toghermore House, Tuam Mental Health Campus, Tuam, County Galway, Ireland; ^2^School of Nursing and Midwifery, National University of Ireland, Galway, Ireland

## Abstract

This study explored registered psychiatric nurses' (RPNs') interactions and level of empathy towards service users with a diagnosis of borderline personality disorder (BPD). A qualitative approach was used, and 17 RPNs were interviewed using a semistructured interview schedule incorporating the “staff-patient interaction response scale” (SPIRS). Four themes emerged following data analysis: “challenging and difficult,” “manipulative, destructive and threatening behaviour,” “preying on the vulnerable resulting in splitting staff and other service users,” and “boundaries and structure.” Additionally, low levels of empathy were evident in the majority of participants' responses to the SPIRS. The findings provide further insight on nurses' empathy responses and views on caring for service users with BPD and further evidence for the need for training and education for nurses in the care of service users diagnosed with BPD.

## 1. Introduction

Mental health nurses frequently care for service users with a diagnosis of borderline personality disorder (BPD) in both hospital and community settings. The literature suggests that BPD is the most prevalent of all personality disorders [1] with an estimated 2-3% of the population meeting the diagnostic criteria [2]. International research indicates that BPD has a higher incidence of occurrence than schizophrenia or bipolar disorder. It is estimated that between 10 percent of service users in outpatient clinical settings and 15 to 20 percent of those in inpatient psychiatric settings meet the diagnostic criteria for BPD [3].

Suicidal or self-harming behaviour is one of the core diagnostic criteria in DSM IV-TR for BPD, and management of and recovery from this personality disorder can be complex and challenging [4]. Suicide rates among those diagnosed with BPD are approximately 8 to 10 percent [5, 6]. BPD is also characterized by service users having a pattern of unstable and intense interpersonal relationships, affective instability, poor impulse control, and self-mutilating behaviour. Many professionals find these service users difficult to interact with, treat and show empathy towards, perhaps because BPD behaviours may aversely effect interpersonal relationships, including relationships with nursing staff [2]. Derogatory terms to describe persons with BPD such as “difficult,” “dangerous,” “treatment resistant,” “manipulative,” “demanding,” and “attention seeking" are often used [7].

A number of studies have examined attitudes of nurses towards service users with BPD [8–15]. These studies report nurses' perceptions of service users with BPD being powerful and destructive in their behaviours. The literature also describes the ability of persons with a BPD to split staff and display manipulative behaviour [7, 11, 15]. In addition, it is reported that service users with a BPD tend to evoke fewer relaxed feelings and quite a high level of aggressive feelings in staff [16]. In contrast, nurses are more likely to respond with sad and self-critical feelings towards psychotic service users and with warm and helpful feelings towards those with neurosis [16]. Other findings suggest that nurses may perceive service users with a BPD to have a greater degree of control over the negative behaviours they display, when compared to those with other disorders [12, 14].

Over recent years there has been some focus on the lived experience of those diagnosed with BPD [17–23]. Women diagnosed with BPD have reported feeling that they were living with a pejorative label, with self-destructive behaviour perceived as manipulative, and having limited access to care because of this [20]. According to the participants in one study [20], health care providers held preconceived and unfavourable opinions of people with BPD, and they referred to their experience as been labelled, not diagnosed [20]. Some service users have spoken about being terrified of disapproval or rejection, particularly from key professionals such as their therapist, and frequently withheld information to defend against this [19]. This sense of being judged negatively by professionals is also reported elsewhere [18]. In terms of living with the diagnosis, service users have described the hopelessness and misery they felt as well as the role of self-harm as a sort-term intervention employed to release pent up emotions and tensions [21]. Those with BPD also report the service being reluctant to give them the diagnosis with two participants in one study only being told their diagnosis when they were recruited for the study [21]. The perception of service users that there is a reluctance to diagnose them with BPD is also reported elsewhere [24].

A number of studies [18–21, 24] recommend that health professionals examine their own attitudes and beliefs surrounding self-harm and BPD and that they engage in meaningful, therapeutic dialogue with these service users. To improve the management and care outcomes for these service users, there is general consensus on the need for more training for mental health professionals [10, 12–14].

There is little published exploring the empathic interactions of psychiatric nurses towards service users with BPD. However, a recent study reports that nurses scored lower than psychiatrists and psychologists on empathy towards those with a BPD [25]. In addition, nurses are reported to have the lowest self-ratings on empathy towards those with BPD when compared to other mental health clinicians [26]. However, considerable work on this topic has been undertaken on the responses of nursing staff to service users diagnosed with a BPD and also the characteristics and stereotypes of service users with BPD [24, 27–31].

There is no “gold standard” tool to measure empathy in the nursing context [28]. It is suggested that one way to measure empathy is to consider the verbally expressed empathy of the nurse, and this approach was undertaken with a group of nurses (*n* = 113) using the staff-patient interaction response scale (SPIRS) [27]. The SPIRS was developed from theoretical views of therapeutic empathy as a multiphase time-sequenced process [32]. This view of empathy as a process was first proposed by the German philosopher Edith Stein (1917–1970) and is one that combines the philosophical, psychological, aesthetic, and the interpersonal [33]. It is described as a three-level model of empathy where a field of tension between views on closeness and distancing in relationships is evident, and sympathy is considered part of empathy [34]. This empathy three-phased process is dependent upon the nurse being attentive to expressed meanings and interpretations that service users attribute to their experience [32]. Therefore, mediators such as nurses' knowledge-beliefs, service users' age, and contextual variables such as hospital site may influence outcomes of each phase of the empathic process.

The SPIRS focuses on phase 2 (expressed empathy) of the three-phase process of empathy [27]. The scale uses the written responses to hypothetical patient stimuli to assess the expressed empathy of staff towards service users diagnosed with BPD and schizophrenia. Responses are scored on a ten-category response scale. The ten categories represent an ascending hierarchy of expressed empathy. The ten scoring categorises represent three levels of empathic care, which are (1) no care, (2) solution, and (3) affective involvement [27]. Using the SPIRS and semistructured interviewing, the study described here aimed to explore registered psychiatric nurses' interactions with and empathy towards service users with a diagnosis of BPD in their care. The specific objectives of this study were to (i) identify common themes from an analysis of the nurses' reported interactions with service users diagnosed with BPD and (ii) describe the level of empathy of RPNs towards service users with BPD using the SPIRS.

## 2. Methods

A qualitative approach was adopted. Qualitative descriptive designs facilitate in-depth interviewing and yield rich participant narratives [35]. The study was undertaken with nurses working in an Irish mental health community service. Inclusion criteria for the study were that participants had to be registered psychiatric nurses with a minimum of three-year postregistration experience, working in a mental health setting for a minimum of two years, and experience of working with service users diagnosed with BPD.

Following ethical approval, a letter was sent to each registered psychiatric nurse meeting the inclusion criteria (*n* = 31) working in the community mental health service inviting their participation in the study. Seventeen nurses contacted the first author (twelve females and five males) and agreed to take part in the study. The nurses' mean years of nursing experience were fifteen years. Six participants worked in a psychiatric community day setting, one worked as a community psychiatric nurse (CPN), and the other eleven participants worked in a community psychiatric residential setting. Eleven participants had obtained a higher-qualification after registration in mental health nursing, and one participant had a masters degree. One participant had undertaken specific training on BPD after registration (a one day workshop). Ten participants reported that they had daily contact with a service user with a diagnosis of BPD. All these were working in a community residential setting. Four reported that they had contact with service users diagnosed with BPD two or three times a week, and a further three reported that they had contact with these service users less than 5 times within a month (these were staff working in community day settings).

Verbal and written consent was provided by each participant before they were interviewed. The first author undertook all the interviews. The first author has ten years postregistration experience in the discipline of psychiatric nursing and works in a community mental health setting within the service where the study was undertaken.

A semistructured interview guide was used which explored participants' interactions and experiences of caring for service users with a diagnosis of BPD. The semistructured interview was followed by questioning participants using the SPIRS [27]. Permission was given by the original authors to use the instrument. Although developed as a questionnaire, the scale was used in the study here to describe the level of empathy for service users with BPD expressed by the participants. The participants were asked to provide their typical response to the scenarios posed in the SPIRS (Boxes 1 and 2).

All seventeen interviews were recorded and transcribed verbatim. Each participant was assigned a pseudonym. Data analysis incorporated two methods of thematic analysis [36, 37] for the responses to the open questioning. A deductive approach was employed to explore the level of empathy expressed by participants to the questions asked using the SPIRS [27].

Following analysis, all the participants were asked to read their transcribed interview for accuracy and to ascertain if the identified themes accurately reflected their views. When returning to the participants after analysis of the data, some participants expressed surprise and concern regarding their level of empathy. Two participants requested for a line to be removed because they were uncomfortable with their responses.

Following thematic analysis of the open questioning, four themes were identified: (1) “challenging and difficult,” (2) “manipulative, destructive and threatening behaviour,” (3) “preying on the vulnerable resulting in splitting staff and other service users,” and (4) “boundaries and structure”. The majority of nurses' responses to the scenarios presented in the SPIRS were at level one (no care) and level two (solution) empathy.

## 3. Findings

The theme “challenging and difficult” reflected the participants' description of their experiences and attitudes in care delivery to service users with a BPD. All participants relayed how challenging and difficult it was to deliver a “good” level of care to those with BPD due to previous experiences with other service users similarly diagnosed. This is demonstrated in the following viewpoints from Helen:

“…*It is difficult because you try to be open and non judgmental and give them the opportunity to take responsibility for their actions and improve their circumstances but they usually sabotage things and take no responsibility blaming others or life events for things not working out.” *

Jane expressed similar views as Helen, saying that persons with a BPD are:

*“Totally difficult patient to manage.. how would I say that… .they are totally self obsessed… . .manipulating you and also they always seem to exaggerate their feeling… .”*

Many of participants' comments were related to the inappropriate behaviour or symptoms that those with BPD display. Yvonne described service users with a BPD as*….*“*difficult to deal with a lot of behavioural problems.”*

Two participants established that they were *“dealing with one behaviour” *or *“symptom focused approach*” to care and this was distracting attention from the true emotional or psychological difficulties of service users with a BPD. While the majority of participants expressed the view that it was not the service user's personally but their behaviour that they found challenging; most also acknowledged that service users with a BPD do not take responsibility for their behaviour.

“*People with attention seeking behaviour and a lot of the time they have unresolved issues and they largely take this out on everyone else”* (John).

“…*they usually refuse to accept the diagnosis alone without stating there are other diagnoses such as an underlying mood disorder to which again they can contribute blame for their actions.” *(Helen).

Participants also commented on the attention seeking behaviour of service users with BPD:

“*Sometimes they are trying to be on your right side all of the time… saying the right things and looking for attention. Other times just trying to get your back up and always trying … . Trying your patience all of the time” *(Mary).

“…*they can give a staff member.. What I call Hedgehog syndrome.. it can get your back up… oh here we go here comes another BPD…you know they all come out with the same kind of stuff but you know maybe different ways..” *(Emma).

Two participants stated that they would avoid providing a service user with BPD any level of care or just a minimal level. Furthermore, they stated that they would avoid any interaction with the service users with BPD until it was completely necessary and they would do this at the end of the day where they knew that there would be no time to explore the issues in depth.

Emma's views, however, were more questioning than other participants:

“…*they do not present with BPD; they present with anxiety or an emotional disorder than any thing else.. Or maybe after any episodes of self harm or maybe referred in from A&E after an over dose.. so.. em.. to describe them… em… they can be a challenging group of clients.. However they are all individual…and I suppose you do get a glance of the real person underneath all that once the crisis has passed a bit. I suppose BPD carry a bit of baggage with them … I suppose that.. they arrive at the service and that they have a diagnosis of BPD it is like they have a red light flashing.. on them… and I suppose.. whatever that brings up for the health professional in question and I suppose, it always brings up your association with previous clients that you have had with BPD*”.

Similarly Lorraine stated that she gained a better insight into herself after her encounters with service users with BPD:

“…*that I am not saying that they are easy to deal with but you have to have a good level of understanding and insight about yourself.. you know that you are not reacting to people all the time. You know if someone says they are going to kill themselves.. you have to realise that it is not about you it is about them,. So you will have to realise I can I help them…*.*” *

The theme “manipulative, destructive and threatening behaviour” reflected the views of all participants who used the terms “manipulative,” “destructive,” and “threatening.” All the participants associated manipulation with the idea that all service users with a BPD have a “hidden agenda” and they try to find out the real agenda behind their actions. Helen describes her interactions with service users who have a BPD “*as superficial and calculated in order to get their needs met by staff.”*

A few participants referred to the term manipulation because they found some service users with BPD to be dishonest and not genuine in nature. Most participants admitted that being manipulated brought about feelings of frustration and being used.

*“Some can be manipulating in behaviour, like playing one nurse off another by making a request to one nurse which is turned down due to policies/care plan in place and then telling another nurse that this request was granted in the first place” *(Kate).

“…* yeah.. well I suppose you just feel so used when they do this to you… as they have used you to get what they want and when that happens you sometimes.. well..em..I suppose you might question your profession? Which leaves you feeling so frustrated as they rope you in …chew you up and then spit you out…” *(Lorraine).

Most participants described service users with a BPD as destructive and threatening. The term “threatening” was used as an umbrella term to describe self-harm or threats of causing harm to other people or property if their needs are not meet. Fifteen participants reported that they found the threat of suicide as the most distressing of all behaviours.

“*Well in any treat of self harm is stressful on the nurse and patient but I mean has to be taken seriously regardless if they have a diagnosis of BPD*” (Emma).

“*You give them time, support and encouragement and in turn they usually continue with behaviours such as deliberate self harm, threatening suicide or absconding. It's difficult to build a therapeutic rapport as their behaviours and actions are manipulative and attention seeking” *(Helen).

Mary described an incident where a service user with a BPD engaged in this type of behaviour and how it caused her personal stress:

*“Someone with BPD was having an argument with another patient. I intervened I asked the person with BPD to keep their voice down. They totally am… ignored me first of all… then they decided to turn all their anger and aggression on to me and I ended up am pinned up against the wall… I then.. set off the alarm.. the personal alarm… the more staff that came to help the BPD person enjoyed it more… seem to enjoy it more got more aggressive and angry and.. acted and play out. The whole situation was a huge learning curve for me and took me a long time to de-stress!”*

Mary also reported that she had received professional debriefing after this incident.

The theme “preying on the vulnerable resulting in splitting staff and other service users” reflected participants' experiences of interactions between service users with a BPD and staff and other service users.

“…*Em.. they will pick out one … weaker one.. get closer to them… initiate relationships which can be inappropriate at times. Staff splitting all the time” *(Jane).

“*They usually lean on people that they believe that are vulnerable and weak be it staff or patients and they play one off the other again.. you know giving conflicting reports and thoughts…” *(John).

Mary described service users with a BPD interactions with other service users:

“*Well at the beginning the other patients believe that the BPD patient is very nice, pleasant, very helpful, getting involved in their care giving advice.. telling them what they should and should not do… . And then suddenly there will be a big bust up.. fighting and arguing and not getting on … splitting of patients and different groups”. *

This brought about tension and stress within staff members:

“…* you will always know who is on duty because the two BPD will not leave the bloody office because they know they can get what they want from X..when they are on.. This is so frustrating because what is the point of a plan when they just give into the demands*” (Bernie).

Lorraine reported a feeling of anger, frustration, hurt and disappointment when she described an incident where a staff member had “given out” to her for doing a task for a particular service user with BPD:

“*I am not weak, How dare they think I am… I was just doing my job and sticking to the plan… . Do they not realise that?*” (Lorraine).

Two participants reported a feeling of paranoia and feeling of mistrust due to service users with a BPD telling them that the opposite shift was “giving out about them.” They spoke about how it was difficult to confront a colleague about the accusation made. Also they spoke about how they became distrustful of the service user with a BPD and their colleagues. This resulted in an unpleasant working environment at times.

The final theme of “boundaries and structure” referred to participants' need for strict boundaries and firm limit setting when interacting with these service users.

“…*at the beginning you can be drawn in or sucked in by someone with BPD and it is only from experience and from dealing with people that you find out that you have to be very strong and that all the staff will have to have a set programme and everyone has to follow that programme” *(Mary).

Emma, however, explored reasons why service users with a BPD have no boundaries.

*Well certainly with the BPD they have poor…Em… uh… ego control they have no sense of boundaries.. BPD they are a high percentage of them would be subjected to some sort of abuse.. emotional, sexual, physical or psychological abuse so I.. They never were kinda… conditioned into boundaries.. so they don't know their own boundaries so therefore they kinda very much infringe in another people's boundaries.. And they don't know they have no concept of themselves like you know! They don't know where their problems end and someone else's problems start. They don't know where their emotions cut off from someone else…so when they are upset they expect you to be equally upset…they don't empathise very well and they don't when you give them empathy they can see that this is very dismissive they are looking for a lot of sympathy! *(Emma).

Some participants spoke about the safety of other service users if service users with a BPD were in the unit for a long admission. Lorraine commented on one particular experience and how distressing it was for staff and other service users:

“*When they are in hospital for ages, they teach other service users harmful behaviours… I saw one BPD teaching a young girl of 18 how to cut herself so she could get attention.. How was that benefiting either of them? We separated them, put a strict rule in place but that just seemed to make them want to get together more! All staff at some point gave up with them.. it was like banging your head off a wall!!” *(Lorraine).

Analysis of the participants' responses to the scenarios presented to them in the SPIRS (Boxes 1 and 2) involved scoring their responses on a ten-category response scale. The scale represents an ascending hierarchy of expressed empathy: (1) no care, (2) solution, and (3) affective involvement (Box 3).

The majority of participants' responses to the questions asked in the SPIRS scenario regarding a first admission (Box 1) offered responses categorised as level one (no care) and level two (offer solution) empathy. A typical category 3 response which explains why rules or processes take place each day is represented by Rebecca's statement:

“*I would explain to her that we work as a team and am.. that there is primary nursing and that you will be assigned a nurse each day … so that they will give you time so you can talk about your worries and concerns*” (Rebecca).

Emma was the only participant whose responses reflected level three empathy to the questions posed in the first scenario. Emma responded by addressing the self-esteem of the girl:

“ …*well what it means to her for me to be listening to her.. So I would look at it what does it mean for her to be listened to.. and what is her association with that you know.. when before did she ever feel listened to”* (Emma).

*“I would take that as a precursor to someone being angry and I would certainly … ask them their feelings.. and go through it with them” *(Emma).

Participants' responses to the second scenario (multiple admissions) presented to them were mostly in the level one empathy. For instance, Jane responded by telling the service user about the rules:

*“I am the nurse on duty and these are the rules… again limits are set….if you are her key worker then you will deal with her appropriately”* (Jane).

To H's statement in the scenario, *“Stop bugging me. Why do you keep trying to talk to me anyway”, *most responses were categorised in category 2. Jane responded by saying “*Again I will ignore her.”* Participants explained that their response to the service user was prompted by their view that he was displaying attention seeking behaviour.

Emma was the only participant whose responses reflected empathy at level three (affective involvement). Her response to the question posed by “H” *“Why should I get dressed—there is no place to go” *invited care and concern:

*“Maybe they didn't want to get dressed. I would ask them to sit out anywhere but the bedroom and I would make a cup of tea for them and talk to them*” (Emma).

The majority of participants offered responses in category 2 (platitudes, clichés, or rules). A typical category 2 response is where the nurse tells the service user about the rules as Jane did when she answered *“They need strict guidelines…this is your plan for the day.”*

The participants scored the highest level of empathy for the last statement asked in both scenarios (*“I wish staff would just let me kill myself—that's the most helpful thing they could do”* Box 1 and *“You have no idea how I feel. I wish I were dead and what can you do anyway?* Box 2). The majority of responses invited exploration with the service users in response to these statements. The participants stated that they would respond in this way because any threat of self-harm has to be taken seriously regardless if they have a diagnosis of BPD or not.

Nine participants did not change their overall level of empathy from the first scenario (first admission: Box 1) to the second scenario (multiple admissions: Box 2) (Table 1).

## 4. Discussion

In harmony with the existing literature, the participants of this study perceived service users with BPD in a negative manner [11, 15]. As discussed earlier, these service users are “challenging and difficult” to deliver care to [8, 11, 15, 38, 39], and this theme was also echoed by the participants of this study. Nurses' feelings of being used and devalued in their experiences are also reported elsewhere [11].

The term “honeymoon to chaos stage” is used to describe nurses' experience of caring for those with BPD in which at the initial stage of treatment it is like a honeymoon where the relationships between service users and nurses were peaceful as they were getting to know each other [15]. However, this is for a brief time frame only and is followed by the “chaos stage” [15] where service users begin to demonstrate various disruptive behaviours, annoying nurses.

Nurses admit feeling tempted to abandon positive expectations for care outcomes at this “chaos stage” [15]. They also report both positive and negative care expectations as well as both positive and negative care outcomes [15]. Similar to the findings in this study, where some nurses admitted providing minimal care and ignoring or avoiding service users with BPD, it is also reported elsewhere that some nurses withdraw and distance themselves from service users with BPD [9]. The level of care provided by nurses to service users with BPD is also questioned [15].

The level of empathy expressed by the study participants for the first admission of a service user with BPD was categorised overall at level 2 (offer solution). With the scenario of multiple admissions, the overall score was lower, categorised at a level one score (no care). These findings are similar to those reported elsewhere where the SPIRS was used [27], and nurses displayed a low level of empathy for service users with BPD in comparison for those with schizophrenia.

Participants who expressed level 1 empathy responses may believe this response is needed because they believe the behaviour of the service user BPD is manipulative or dangerous. It is reported that nurses believe it is socially acceptable to respond to the service user with BPD this way because of their diagnosis [14]. Service users with BPD are known to create a myriad of feelings among nurses. These include feelings of helplessness, being used, therapeutic failure, devalued and unappreciated, anger, and frustration [11, 15]. Therefore, this level of response may be a defence mechanism against the intense affect generated in nurses by service users with BPD.

The interview data in this study reveals that participants have a clear understanding of the characteristics of service users with BPD. Almost collectively they agreed that those with BPD were difficult and challenging to provide care for. The majority of participants also believed that service users with BPD should not be cared for in a hospital environment because the services are inadequate and this has a negative impact on care delivery. The development of a specialist community service and improved education and skills training workshops were viewed by the participants as being the most probable way to improve the service and care received by service users with BPD. These service users' behaviours are complex and arise from many causes, biological, psychodynamic, and sociocultural, which all work together to create the behaviours. Therefore, nurses need to understand both the origins and the functions of these problems [40]. The more the nurses understand the complexity of BPD, the more likely a higher level of empathy may be displayed. Therefore, it may become less demanding for the nurses to respond therapeutically and consistently without anger, frustration, and fear of service users with BPD.

A number of suggestions have been proposed about the content of such training; for example, one study recommended cognitive behavioural therapy because of its perceived evidence base [11]. The development and delivery of a brief training workshop on BPD for public mental health clinicians is reported in [41]. The aim of the workshop was for participants to develop practical skills in carrying out treatment plans and to become more positive about working with service users with a diagnosis of BPD. It was thought that this would also improve relationships between service users with BPD and nurses by helping nurses to overcome negative perceptions that they associate with these service users. After the workshop, the participants' confidence and willingness to work with people with BPD had statistically improved. It was also reported that the workshop provided a forum to improve understanding of the challenges and complexities faced in different work settings [41]. Practice guidelines set out also recommend supervision for staff caring for individuals with BPD [3]. With appropriate training and support, nurses can be educated about realistic expectations of treatment outcomes to counter later pessimism that may arise. Addressing these issues can modify negative nursing responses and help alleviate negative working experiences with service users with BPD [14]. Nurses need to receive regular supervision; this will provide them the opportunity and space to process any perceived unpleasant experiences generated from caring for service users with BPD and help prevent burnout [42].

In conclusion, a better understanding of the complexity of BPD may help nurses to respond therapeutically and consistently without anger, frustration, and fear. Service users with BPD symbolise a challenge to nurses; however, with improved education, training, and clinical supervision, a new era in BPD treatment can begin [43]. Nurses who view these service users in a holistic manner can frame research and practice in a way that can positively affect these service users' lives [21, 44]. Nurses who hold positive attitudes about service users, have a sense of moral commitment, are skilled interpersonally, and are able to stay in a rational state of mind in the midst of conflict and can apply knowledge about the personality disorder and work skillfully with these service users [45].

Although the study findings add further evidence to suggest the need for training and education for nurses caring for service users with BPD, the study findings are limited for a number of reasons. The participants all work in one mental health service area and including nurses from other mental health service areas may have yielded different findings. The sample was self-selecting, and those who came forward for interview may have had a greater interest in BPD than those who did not. Moreover, it is possible that the views of those who did not choose to take part in the study may differ from those who were interviewed. Finally, social desirable answering may have been a factor in some of the nurses' responses.

## Figures and Tables

**Box 1:**
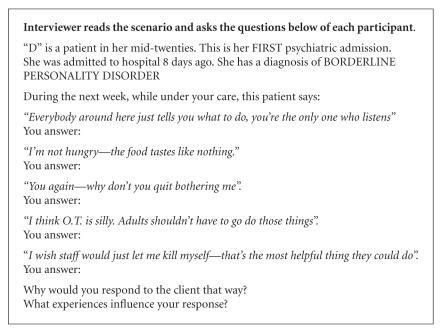
SPIRS scenario (first psychiatric admission) [27].

**Box 2:**
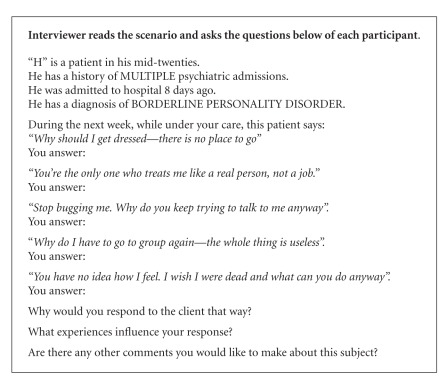
SPIRS scenario (multiple psychiatric admissions) [27].

**Box 3:**
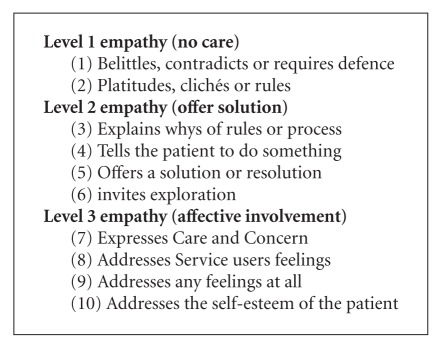
Levels of empathy (SPIRS) [27].

**Table 1:** Average level of empathy across all levels of empathy and scenarios.

Participant	First-admission level of empathy displayed	Multiple-admission level of empathy displayed	Change in empathy level
	Level 1: no care Level 2: offers solutions Level 3: affective involvement	Level 1: no care Level 2: offers solutions Level 3: affective involvement	
Helen	Level 2	Level 1	↓
Kate	Level 2	Level 1	↓
John	Level 2	Level 1	↓
Tiberius	Level 2	Level 2	*↔*
Anna	Level 1	Level 1	*↔*
Jane	Level 1	Level 1	*↔*
Jimmy	Level 2	Level 1	↓
Emma	Level 3	Level 3	*↔*
Susie	Level 2	Level 1	↓
Rebecca	Level 2	Level 1	↓
Bernie	Level 2	Level 2	*↔*
Paris	Level 1	Level 1	*↔*
Yvonne	Level 1	Level 1	*↔*
Mary	Level 2	Level 1	↓
Fergal	Level 1	Level 1	*↔*
Brendan	Level 1	Level 2	↑
Lorraine	Level 2	Level 2	*↔*
